# Inhibin subunit beta A promotes cell proliferation and metastasis of breast cancer through Wnt/β-catenin signaling pathway

**DOI:** 10.1080/21655979.2021.1971028

**Published:** 2021-12-10

**Authors:** Tao Xueqin, Mei Jinhong, Huang Yuping

**Affiliations:** Department of Pathology, The First Affiliated Hospital of Nanchang University, Nanchang, Jiangxi Province, China

**Keywords:** INHBA, proliferation, EMT, Wnt/β-catenin pathway, breast cancer

## Abstract

Emerging evidence has demonstrated that inhibin subunit beta A (INHBA) is dysregulated and plays a critical role in various cancers. With the development of sequencing technology, studies have discovered that INHBA is overexpressed in breast cancer tissues. However, the biological roles of INHBA in breast cancer are still far to clear. In the present study, we analyzed the INHBA expression in the Cancer Genome Atlas (TCGA) database. Quantitative real-time polymerase chain reaction (qRT-PCR) was conducted to assess the expression of INHBA in breast cancer cell lines. Cell proliferation, invasion and epithelial–mesenchymal transition (EMT) were determined by using CCK-8, EdU, Transwell and western blot assays. The result showed that INHBA was highly expressed in breast cancer cell lines. Functional analysis revealed that silence or elevation of INHBA inhibited or promoted the proliferation, migration, invasion and EMT and Wnt/β-catenin signaling pathway-related markers of MCF-7 cells. Mechanically, blocking of Wnt/β-catenin pathway by XAV939 reversed the promotion effect of INHBA overexpression on breast cancer cells’ proliferation, migration and invasion. Our findings emphasized that INHBA may act as an oncogene via activating the Wnt/β-catenin pathway, which may provide a potential therapeutic target for the treatment of breast cancer.

## GRPAHICAL ABSTRACT

**Figure:**
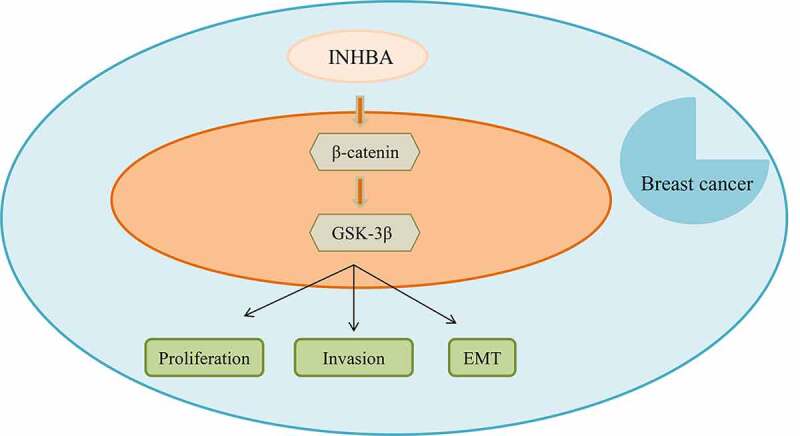

## Introduction

Breast cancer is one type of the most common malignant tumors in women worldwide [1,2]. The incidence of breast cancer has increased annually in recent years. With the continuous improvement of diagnosis and treatment, including chemotherapy, radiotherapy, fungus and molecular targeted biomarkers in the last few decades, the mortality of breast cancer patients remains relatively high [3–6]. Hence, it is urgent to elucidate the molecular mechanisms underlying the initiation and development of breast cancer.

INHBA A is located at 7p14.1, which is a member of the transforming growth factor β (TGF-β) superfamily [7]. Many studies have proved that INHBA serves important roles in various cancer progressions. Peng et al. discovered that INHBA knockdown inhibits nasopharyngeal carcinoma cell proliferation and invasion of SUNE1 [7]. INHBA gene silencing has been reported to be able to inhibit cell migration and invasion by TGF-β signaling pathway in gastric cancer [8]. In addition, INHBA has been demonstrated to be a prognostic predictor for colon adenocarcinoma patients [9]. More importantly, Wang et al. found that INHBA was upregulated in the breast cancer tissues [10]. However, the function and potential mechanism of INHBA in breast cancer remains unclear.

In the present study, we also found that INHBA is simultaneously over-expressed in breast cancer tissues and cells. We then focused on exploring the functions of INHBA in the progression of breast cancer and demonstrated that inhibition of its expression could markedly attenuate the proliferation and epithelial–mesenchymal transition (EMT) of breast cancer cells. Mechanistically, we elucidated the mechanism of INHBA in the breast cancer progression and indicated that it could regulate breast cancer cell invasion and EMT through Wnt/β-catenin signaling pathway. Our findings illustrate a new target and underlying mechanisms of breast cancer progression, which provide an effective target for the treatment and diagnosis of breast cancer, and extend the understanding mechanism-related functions of INHBA in breast cancer.

## Materials and methods

### Cell culture and treatment

MDA-MB-468, SUM149PT, MCF-7, SKBR3, MCF-10A cells were purchased from the Type Culture Collection of the Chinese Academy of Sciences (Shanghai, China) and routinely cultured in Dulbecco’s Modified Eagle’s medium (DMEM, Invitrogen, Carlsbad, CA, USA) supplemented with 10% fetal bovine serum (FBS, LifeTechnologies, Inc., USA) in a humidified cell incubator at 37°C with 5% CO_2_.

### Cell transfection

Cells were transduced with lentivirus targeting INHBA (sh-INHBA) as well as their corresponding negative control, INHBA overexpression vector (INHBA) and empty vector (pcDNA3.1) were purchased from GenePharma (Shanghai, China). For transfection, cells were plated into six-well plates and transfected using Lipofectamine 2000 (Invitrogen, Carlsbad, CA, USA) according to the manufacturer’s protocol. The sequences were used as follows: INHBA shRNA-1: 5′-GCTTCTGAACGCGATCAGAAA-3′, shRNA-2: 5′- AGGCACTTTCCTACCCAATTA-3′, shRNA-3: 5′- CCAACAGGACCAGGACCAA-3′.

### Cell counting kit 8 (CCK8) assay

Cell viability was detected by MTT assay. MCF-7 cells (1 × 10^5^ cells/well) were seeded in 96-well plates and cultured for 24 h, 48 h and 72 h. Then, cell viability was examined by CCK-8 kit (Beyotime Biotechnology, China). In brief, 50 µL of CCK-8 solution was added into each well and incubated at 37°C for 4 h. The absorbance value was measured by a microplate reader at 490 nm wavelength. The experiment was repeated three times.

### Transwell assay

Cells were collected and plated in the upper chamber (Corning) coated with (invasion) or without (migration) Matrigel (0.1%, Millipore, MO, USA). 1 × 10^5^ cells in serum-free medium in the upper chamber of the transwell. A culture medium containing 20% FBS was supplemented into the lower chamber. Following 24 h of incubation, the non-migrated and non-invading cells were removed. At last, cells were fixed with 4% formaldehyde and stained using crystal violet. Cells were counted under a microscope (Olympus, Tokyo, Japan) at 200× magnification. The number of cells was the average value of six representative fields. The migratory or invaded cells were counted and photographed under a light microscope (Nikon, Tokyo).

### Quantitative reverse transcription polymerase chain reaction (qRT-PCR) assay

Total RNA extraction was harvested utilizing TRIzol (Invitrogen). RNA quantification was amplified and detected on ABI PRISM 7900 Real-time PCR system (Applied Biosystems). The reaction conditions were as follows: 94°C 3 min, 94°C 30 s, 56°C 40 s, 72°C 30 s, a total of 32 cycles, and finally extended at 72°C for 10 min. GAPDH used for normalization and the relative expression level was calculated by the 2^−ΔΔCt^ method. The primers were as followed: INHBA: 5′- ACACAACAACTTTTGCTGCC-3′ (Forward), 5′- TCGTGTCACCACTGTCTTCTC-3′ (Reverse).GAPDH: 5′-GAAGGTGAAGGTCGGAGTC-3′ (Forward) and 5′- GAAGATGGTGATGGGATTTC −3′ (Reverse).

### Western blot analysis

Total protein was extracted from cells using ice-cold RIPA lysis buffer (Beyotime Biotechnology, China). The concentration was estimated through a BCA protein assay kit (Beyotime Biotechnology, China). Equivalent samples (20 µg) were separated by 10% SDS-PAGE, and then transferred onto a polyvinylidene fluoride membrane (Millipore, Billerica, MA, USA). Subsequently, the membrane was blocked utilizing nonfat milk for 2 h and incubated with the following primary antibodies overnight at 4°C, including E-cadherin (ab231303, 1: 1, 000, Abcam), N-cadherin (ab76057, 1: 1, 000, Abcam), β-catenin (ab68183, 1: 1, 000, Abcam), p-GSK-3β (ab93926, 1: 1, 000, Abcam), GSK-3β (ab32391, 1: 1, 000, Abcam), GAPDH (ab128915, 1: 1, 000, Abcam). The membrane was then washed three times with PBST. The membrane was then cultured with HRP adjusted second antibody for about 2 h at room temperature. Protein bands were visualized with Enhanced Chemiluminescence Detection Kit (Thermo Fisher Scientific, USA) and the density of the bands was quantified using ImageJ software. GAPDH was used as the loading control.

### Statistical analysis

GraphPad prism 8.0 (GraphPad, San Diego, CA, USA) was applied for statistical analysis. Data are presented as the mean ± standard deviation (SD). Comparisons between two groups were determined using Student’s t-test. One-way ANOVA analysis followed by Turkey’s post hoc was used for multiple comparisons. P < .05 meant statistically significant.

## Results

### INHBA expression was up-regulated in breast cancer

**Figure 1.**
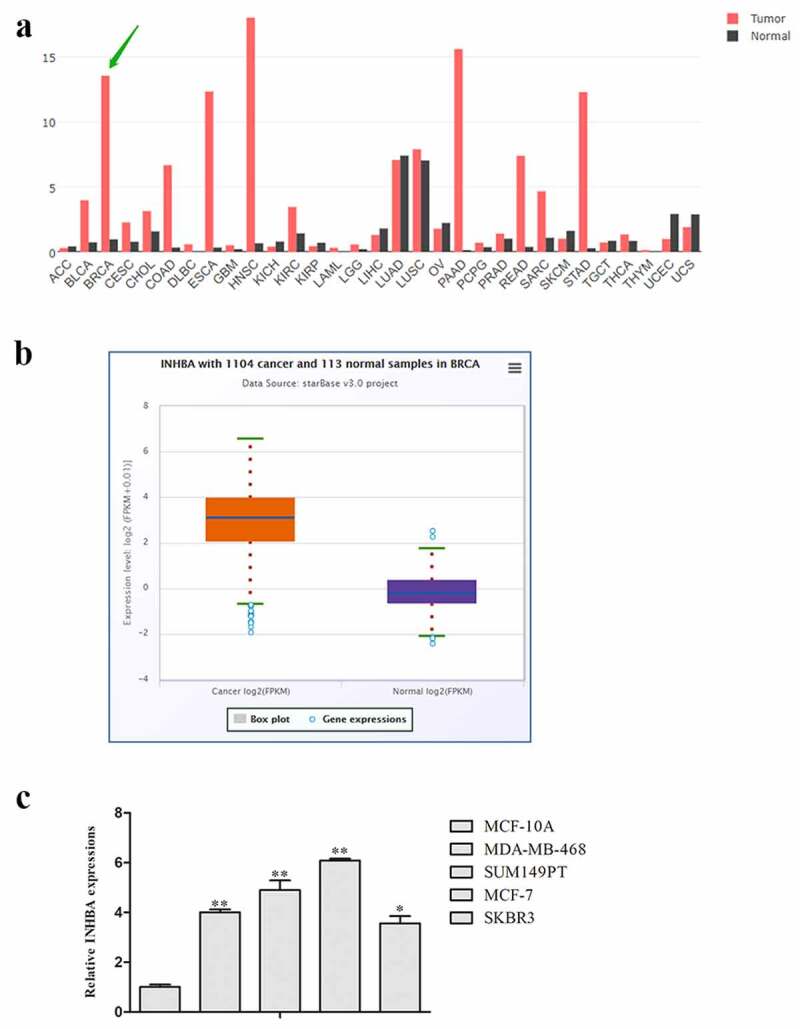
The expression levels of INHBA were up-regulated in breast cancer. A, B. The expression levels of INHBA in 1104 breast cancer tissues and 113 normal tissues in GEPIA database. C. The related expression of INHBA in breast cancer cell lines was analyzed

After browsing the Gene Expression Profiling Interactive Analysis (GEPIA) database (http://gepia.cancer-pku.cn/), we found that the INHBA expression level was significantly higher in BRCA tissues than normal tissues (Figure 1a, b). Subsequently, the expression level of INHBA in BC cell lines and human normal epithelial cell-line MCF-10A was examined through qRT-PCR analysis. Consistently, the results indicated that the expression level of INHBA was dramatically elevated in breast cancer cell lines compared to MCF-10A cells, especially in MCF-7 cells that were used in the subsequent experiments (Figure 1c).

### INHBA knockdown inhibits cell proliferation of breast cancer

**Figure 2.**
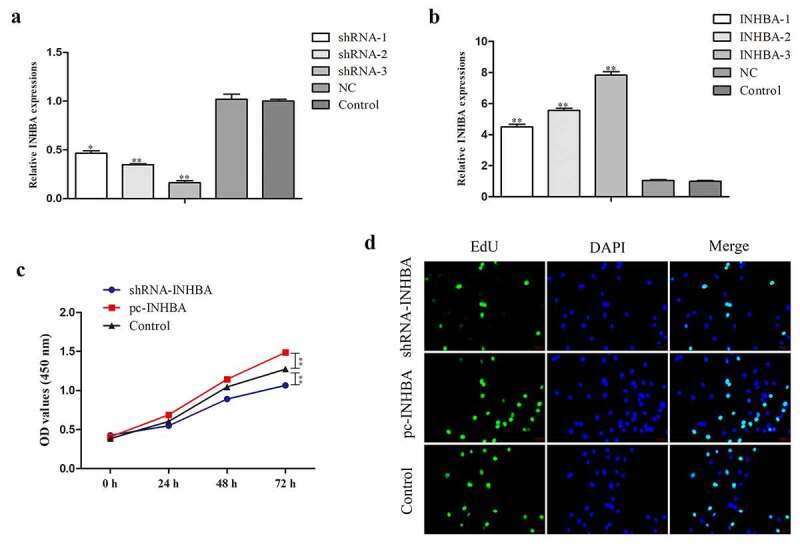
INHBA knockdown inhibits cell proliferation of breast cancer. A, B. The transfection efficacy was determined via qRT-PCR. C, D. The effect of INHBA on breast cancer cell proliferation was evaluated by CCK-8 and EdU assays

To probe the role of INHBA in breast cancer, the INHBA overexpression plasmid and INHBA knockdown plasmid were transfected into MCF-7 cells, in order to up-regulate or down-regulate the INHBA expression. The transfection efficacy was determined via qRT-PCR. Likewise, the data showed that INHBA expression was conspicuously increased in MCF-7 cells transfected with pc-INHBA, while decreased in MCF-7 cells transfected with sh-INHBA relative to NC group (Figure 2a). The efficacy of shRNA-3 and INHBA-3 was better, so we chose shRNA3 and INHBA-3 for the next experiment.

Next, the effect of INHBA on breast cancer cell proliferation was evaluated by CCK-8 assay. As shown in Figure 2b, the result indicated that compared with control groups, silenced INHBA expression led to decreased cell proliferation rate. However, the viabilities of MCF-7 cells when transfected with pc-INHBA was relatively promoted compared with that of the control group (Figure 2c). Similarly, the results of EdU experiment unveiled that INHBA silencing or promotion markedly suppressed or facilitated the proliferation of MCF-7 cells in comparison with the control group. Additionally, INHBA overexpression led to the opposite trend in the ability of MCF-7 cells (Figure 2d).

### INHBA knockdown suppresses the invasion and EMT of breast cancer cells

**Figure 3.**
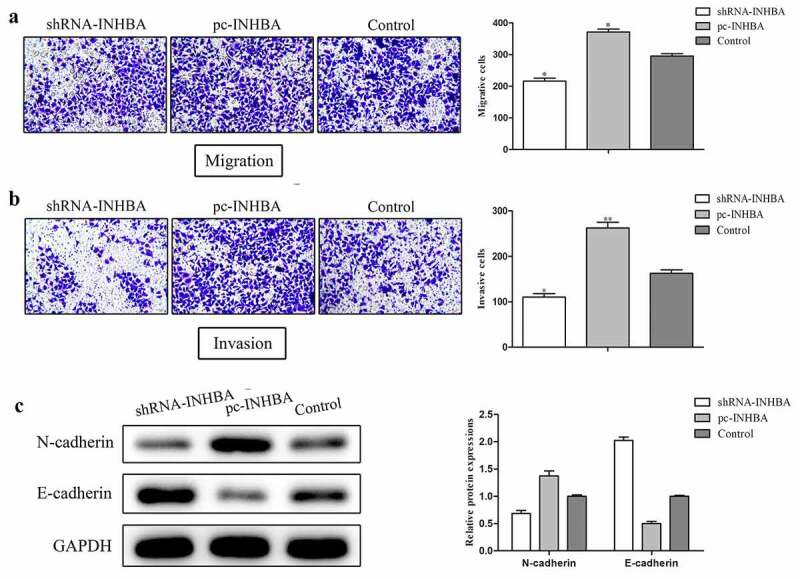
INHBA knockdown suppresses the invasion and EMT of breast cancer cells. A, B. Cell migration and invasion were analyzed by transwell assay. C. Western blot analysis was used to determine the protein level of EMT related markers

Transwell migration and invasion assays were performed to assess the effects of INHBA on the migration and invasion properties in MCF-7 cells. Compared with the control groups, INHBA down-regulation led to inhibition, while INHBA overexpression led to promotion of both migration and invasion of MCF-7 cells (Figure 3a, b). Epithelial-to-mesenchymal transition (EMT) is a process by which epithelial cells acquire the characteristics of mesenchymal cells in tumor progression [11,12]. EMT-related markers containing E-cadherin and N-cadherin were assessed. As shown in Figure 3c, the E-cadherin protein expression level was increased, while N-cadherin expression level was decreased when INHBA was knocking down. Whereas the E-cadherin protein expression levels in pc-INHBA group were lower, while N-cadherin expression level was higher than those in the control group.

### INHBA facilitated breast cancer cell invasion and EMT through activation of the Wnt/β-catenin signaling pathway

**Figure 4.**
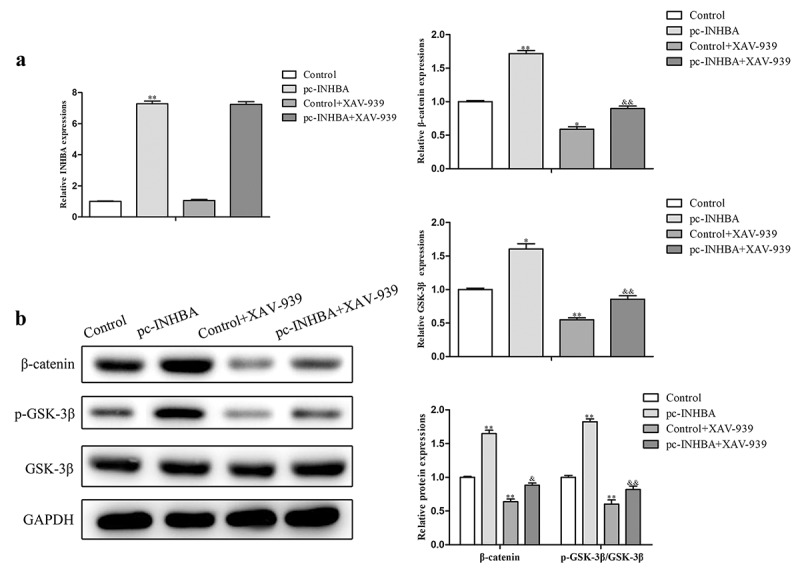
INHBA facilitated breast cancer cell invasion and EMT through activation of the Wnt/β-catenin signaling pathway. A. Wnt/β-catenin signaling pathway associated mRNA and protein expressions were assessed using qRT-PCR and western blot assays

Wnt/β-catenin signaling pathway plays an essential role in cancer progression, and we speculated that INHBA facilitated breast cancer progression through Wnt/β-catenin signaling pathway. To test our hypothesis and investigate the possible mechanism of INHBA in regulating the invasion and EMT of breast cancer, we investigated the expression of the related mRNA and proteins in Wnt/β-catenin pathway. We constructed INHBA plasmid to force expression of INHBA in MCF-7 cells (Figure 4a), and treated with XAV-939 (Wnt/β-catenin signaling inhibitor). Compared with the control group, ectopic of INHBA significantly increases β-catenin and GSK-3β mRNA expression levels and can be reversed by XAV-939 treatment (Figure 4a). As shown in Figure 4b, the overexpression of INHBA group showed an up-regulation in the protein expression levels of β-catenin and phosphorylation levels of GSK-3β. Meanwhile, these effects were reversed by XAV-939 treatment.

**Figure 5.**
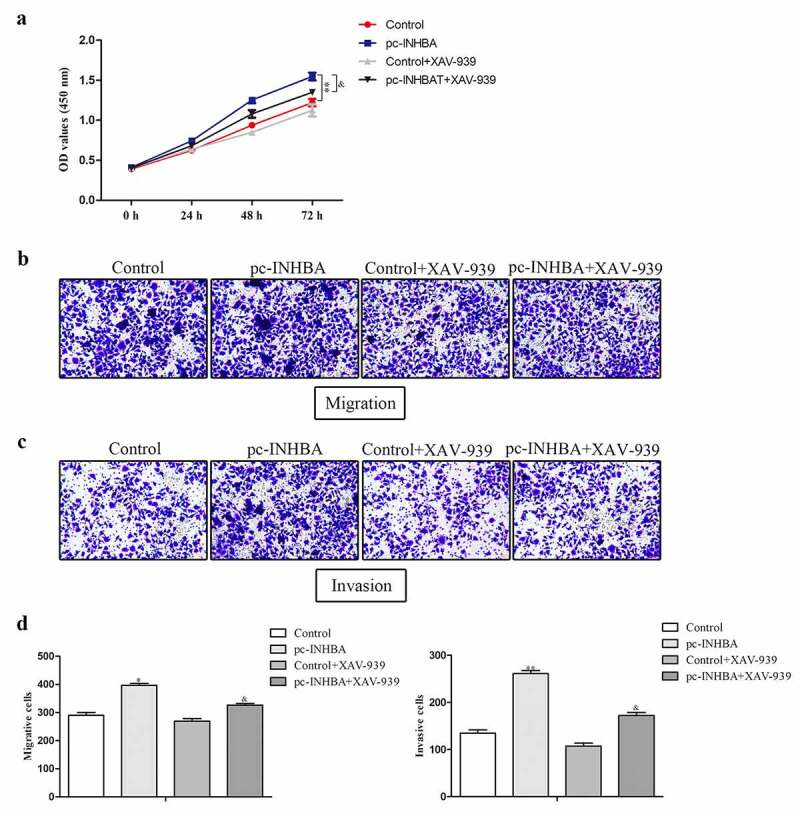
INHBA plays its role in promoting tumor progression through Wnt/β-catenin signaling pathway. A. The proliferative capacity of MCF-7 cell was evaluated by CCK-8 analysis. B-D. The migration and invasion abilities of MCF-7 cells were estimated by transwell assay

To investigate whether INHBA plays its role in promoting tumor progression through Wnt/β-catenin signaling pathway, rescue experiments were performed. Followed functional analysis indicated that over-expression of INHBA facilitated the proliferative capacity of MCF-7 cells, while they were partly suppressed by XAV-939 treatment compared with control group (Figure 5a). In addition, the migration and invasion abilities of MCF-7 cells transfected with pc-INHBA were higher than those in the control group. These effects were reversed in MCF-7 cells treated with XAV-939 (Figure 5b-d).

## Discussion

In recent years, with the development of molecular biology, individualized cancer management has developed rapidly. Breast cancer has become one of the most challenging malignant tumors in women. Interest in finding useful biomarkers for diagnosis and treatment of breast cancer has been accumulating. Increasing data indicated that aberrant INHBA expression has served important roles in almost all cell biological behaviors, including growth, cell cycle, apoptosis, differentiation, apoptosis and metastasis [11–15]. However, little is known about the function of INHBA in breast cancer. The purpose of our study was to explore the role of INHBA in breast cancer.

By performing a series of bioinformatics analyses, we found that INHBA was notably up-regulated in breast cancer tissues. Based on clinical evidence, we deduced that INHBA may affect breast cancer cell functions. Based on the results of qRT-PCR, INHBA expression was higher in breast cancer cells than that in normal epithelial cells. In vitro experiments were performed to evaluate the role of INHBA in cell proliferation and invasion of breast cancer by using the CCK-8, EdU and transwell assays with MCF-7 cells exhibiting overexpression or knockdown of INHBA. As expected, we found that knocking down of INHBA could inhibit cell proliferation, migration and invasion, indicating that INHBA down-expression exerted a significant inhibitory role in these biological processes.

Tumor metastasis involves many tumor processes and EMT is a key initial step for tumor cells to acquire the potential of metastasis and invasion [16,17]. EMT is an evolutionary process in which cells lose epithelial properties and acquire mesenchymal properties. E-cadherin and Vimentin as the epithelial biomarkers have been proved to play an important role in tumor metastasis [18–21]. For example, Zhang et al. found that GRIM-19 could inhibit colorectal cancer cell invasion and EMT by inactivation of STAT3/HIF-1α signaling axis [22]. REC8 have been proved to inhibit gastric cancer cell EMT by down-regulating EGR1 expression [23]. In the present study, the results of qRT-PCR and Western blot assays demonstrated that knockdown of INHBA markedly repressed the expression level of mesenchymal marker N-cadherin, while the expression level of epithelial marker E-cadherin was increased.

The molecular mechanisms by which INHBA affects cancer progression remain elusive. Many studies have focused on metastasis and proliferation of tumor cells themselves. Wamsley et al. found that INHBA has been shown to induce and maintain mesenchymal phenotypes of cancer stem-like cells and to promote cancer cell metastasis in non-small-cell lung cancer [24]. Additionally, INHBA gene is found to mediate activation of the TGF-β signaling pathway to promote gastric cancer cell migration and invasion [8]. However, in breast cancer, the role of INHBA is mixed and has not yet been fully elucidated. The role of Wnt/β-catenin signaling played in the growth and metastasis of tumor has been investigated in many researches [25–27]. The above findings led us to consider the potential association of INHBA with the Wnt/β-catenin pathway. The results indicated that ectopic of INHBA expression activated Wnt/β-catenin pathway with the change of β-catenin and GSK-3β. In addition, the disruption of Wnt/β-catenin signaling by XAV-939 treatment reversed the stimulative effect of INHBA overexpression on breast cancer cell proliferation, migration and invasion.

## Conclusions

Our results demonstrated that INHBA was significantly stimulated the proliferation, migration, invasion and EMT of breast cancer by activating Wnt/β-catenin pathway. These findings indicated that INHBA may be a potential candidate predictive factor in breast cancer, which may contribute to the diagnosis and treatment of breast cancer. I will further investigate the pathogenesis of INHBA in relation to breast cancer and its underlying mechanisms using in vivo model in the future.

## Data Availability

All data generated or analyzed during this study are included in this published article.

## Highlight

- INHBA was significantly stimulated the proliferation, migration, invasion and EMT of breast cancer.
- INHBA plays its role in promoting tumor progression through Wnt/β-catenin signaling pathway.
- NHBA may be a potential candidate predictive factor in breast cancer, which may contribute to the diagnosis and treatment of breast cancer.
